# Puerpural Apoplexy

**Published:** 1882-01

**Authors:** Thomas Robertson


### Art. V.—PUERPURAL APOPLEXY.

*History.*—Parturient apoplexy, milk fever, dropping after calving, etc., is a disease confined almost wholly to the bovine species. England and Germany have lost many of their most valuable cows with this disease, notwithstanding the very best of care and medical treatment to which they were subjected. The animals most subject to this condition are those which have been pampered and fed upon stimulating diet, such as meal, beans, barley and corn, and have had too little exercise. As a rule, the animals most frequently attacked are the pure-bred animals, particularly the Alderneys. From them we can grade down the scale in pretty direct ratio as to liability, beginning with the best bred cows, in the highest condition and heaviest milkers, and ending with those without lineage and in the worst possible condition.

This disease is generally associated with parturition, and is often confounded with a form of paralysis dependent on entirely different conditions.

It is more likely to occur in warm weather than in cold, in good milkers than in poor, and one attack predisposes to another.

It sometimes occurs before labor, but not often. It generally makes its appearance very shortly after parturition, within the first three days, and not, as many suppose, three weeks after.

*Pathology.*—Septic poisoning, indigestion, nervous derangement, anatomical predisposition, metritis, encephalic anæmia and a variety of other conditions have all been assigned as the chief cause of the trouble. Our best authorities, however, ascribe it to disorder of the sympathetic nervous system.

In the milch cow this system is most highly developed. Alimentation, lactation and utero-gestation are under its direct control, and the influences are markedly manifest in the various secretory processes.

During parturition an excess of blood is thrown upon this system. There is sudden increase of blood-pressure, and congestions and apoplectic lesions result. The great volume of blood used for the nourishment of the fœtus in utero by the act of parturition is no longer required in the generative organs, and is cut off. The mammary gland, hitherto inactive, suddenly takes on great activity, accompanied with increased circulation and excessive secreting power. One system of nervous action thus becoming inactive, while another is as rapidly called upon for compensating activity. If any causes tend to prevent this vicarious or safety-valve action, congestion and apoplexy naturally follow in the part involved, which in this case is located in the sympathetic gangliated cord.

*Symptoms.*—The cow generally within a few hours after labor, becomes restless; there is swinging of the hind feet or a stepping action. The animal breathes quickly; is indisposed to move; staggers in walking. The eye is bright and staring, and there is a general appearance about the expression denoting cereberal excitement. She moves her ears spasmodically; there is spasmodic contraction of the facial muscles, grinding of the teeth, and evidences of great prostration. The lacteal secretions are lessened or entirely disappear. The sudden stoppage of the milk is a sign of great significance, and is almost pathognomonic of this disease.

The hind limbs gives way, and the animal falls and remains down. As the disease progresses the eyes protrude and become bloodshot, pupils dilated, and insensible to light, and there is general loss of sensation and voluntary motion. The bowels are obstinately constipated; urine scanty and high colored.

The pulse, in the earlier stage, is full and soft, and occasionally slow; in the later stage it is rapid, small and finally imperceptible; the respiration slow and infrequent, and finally stertorious; head and horns hot; animal frequently delirious, dashing the head about with great violence or becoming finally comatose; it lies with the head flexed on the shoulder.

The power of deglutition is greatly impaired or lost entirely; the bowels cease to act, and the urine is retained. Tympanitis is a constant symptom.

In fatal cases all the symptoms become more marked; the coma is deeper; the vitality lessens, and death closes the scene.

In favorable cases, the internal organs commence to resume their functions; the bowels act; the pulse is stronger; respiration more frequent, and gradually the patient recovers.

Relapses frequently occur. Convalescence is usually slow and, in some instances, there is a decided tendency to sloughing to the parts subject to pressure.

*Treatment.*—The most important consists in prophylactic measures. This consists in keeping up a due amount of exercise, and avoiding all kinds of food which tend to induce plethora.

In high bred animals, it is well to resort early to cathartic remedies (salines), and, if necessary, deplete with the lancet.

If, however, the case is not seen till the disease has already developed a full cathartic should be exhibited. I have found the following useful:

℞

| | |
|---|---|
| Magnesia Sulphus, | ℔ xii. |
| Pulv. Croton Seeds, | No. xxx. |
| Pulv. Zinzeberis, | ℥ ii. |
| Pulv. Gentian, | ℥ ii. |
| Aq. Puræ, | q. s. |

If the patient is in the earlier stages, the abstraction of blood is decidedly beneficial and should be resorted to without delay.

The most important indication is to get the bowels to act. Very large doses are required often, to do this, for there is great tolerance of drugs in this disease.

Careful nursing is an absolute necessity. The patient should be well supported and bedded with clean straw and well covered with cloths, and friction applied all over the surface of the body. All lacteal secretions should be removed regularly. Cold water or ice may be applied to the head, supplemented with full doses of whiskey, two pints every three hours, combined with two ounces of spts. turpentine, and hot water, if there is much tympanitis.

The action of the skin can be stimulated by the use of hot blankets, and the application of stimulating liniments along the spine.

If the power of deglutition is impaired, the medicine should be administered by the aid of the stomach pump. It is well, in those cases which tend to recovery, to confine the patient for some time to a gruel diet. Enemas aid cathartic action and the urine should be regularly drawn off with the catheter.

Resolution is more frequent in some parts of the country than in others, but the disease is peculiarly fatal in any section. During convalescence, tonic remedies are indicated, especially strychniæ and the iodide of potassium. Blisters along the spine are serviceable in cases of persistent paralysis.

The flesh of animals suffering from milk fever is not fit for human food, although there is no evidence that it will cause disease in animals fed upon it.

*Post mortem Appearances.*—Veins distended with dark-colored blood; petechial appearance of serous membranes; congestion of the brain and spinal cord with apoplectic clots in various parts, and occasionally an effusion of serum in the spinal canal, and spinal cord softened.

THOMAS ROBERTSON, M.R.C.V.S.

